# Brief report: Decreased expression of CD244 (SLAMF4) on monocytes and platelets in patients with systemic lupus erythematosus

**DOI:** 10.1007/s10067-017-3698-2

**Published:** 2017-06-08

**Authors:** Anselm Mak, Susannah I Thornhill, Hui Yin Lee, Bernett Lee, Michael Poidinger, John E Connolly, Anna-Marie Fairhurst

**Affiliations:** 1 Department of Medicine, National University of Singapore, Singapore, Singapore; 2 Division of Rheumatology, National University Hospital, Singapore, 119074 Singapore; 3 Singapore Immunology Network, A*STAR, Singapore, 138648 Singapore; 4 Institute of Molecular and Cell Biology, A*STAR, Singapore, 138673 Singapore; 5 Institute of Biomedical Studies, Baylor University, Waco, TX 76798 USA; 6 Department of Microbiology and Immunology, Yong Loo Lin School of Medicine, National University of Singapore, Singapore, Singapore

**Keywords:** CD244, Monocytes, Platelets, SLAMF, SLE

## Abstract

The signalling lymphocyte activation molecule (SLAM) family receptors play important roles in modulating immune responses. Previous studies in murine models and patients have suggested an association of the SLAM family (SLAMF) members with the development of autoimmunity, particularly systemic lupus erythematosus (SLE). Since previous investigations on CD244 expression have focussed on NK and T cells, the aim of this study was to evaluate the surface expression of major SLAMF members across monocytes and polymorphonuclear cells in an Asian SLE cohort and explore their potential associations with SLE-related disease activity and autoantibodies. Thirty-nine SLE patients and twenty-nine healthy controls (HC) were evaluated for the expression of CD150, CD84, CD229, CD48, CD244, CD352 and CD319. We determined a significantly lower expression of CD244 on monocytes in SLE patients compared to HC. Furthermore, monocyte CD244 expression was negatively associated with several serum autoantibody titres. Our findings suggest that this molecule plays an important role in immune tolerance mechanisms and should be investigated further.

**Electronic supplementary material:**

The online version of this article (doi:10.1007/s10067-017-3698-2) contains supplementary material, which is available to authorized users.

## Introduction

Systemic lupus erythematosus (SLE) is a chronic autoimmune disorder affecting predominantly females of childbearing age and characterised by the development of autoantibodies to nuclear antigens [1, 2]. Antinuclear antibodies (ANAs) form immune complexes with self-reactive material which deposit in tissues and promote infiltration leading to tissue destruction [3]. Both genetic and environmental factors have been implicated in SLE onset, with a syntenic region on chromosome 1 in humans and mouse models of lupus associated with disease susceptibility. In lupus-prone mice, the *Sle1b* locus on chromosome 1 mediates loss of tolerance and the development of highly penetrant ANAs [4]. This region encodes the highly polymorphic signalling lymphocytic activation molecule (SLAM) family genes and, in humans, the cluster of SLAM family genes are also located within the 1q23 region linked to SLE susceptibility [5, 6].

The SLAM family of receptors are expressed on cells of haematopoietic origin and play important roles in immunomodulation through predominantly homotypic interactions. The SLAM family of receptors in humans include CD150 (SLAMF1), CD48 (SLAMF2), CD229 (SLAMF3), CD244 (SLAMF4), CD84 (SLAMF5), CD352 (SLAMF6) and CD319 (SLAMF7). Receptor ligation results in signalling through immunoreceptor tyrosine-based switch motifs (ITSMs) which bind small adaptor proteins such as SLAM-associated protein (SAP) and Ewing’s sarcoma (EWS)-activated transcript-2 (EAT-2). In humans, SAP deficiency results in X-linked lymphoproliferative disorder (XLP), associated with impaired humoral immunity and defects in natural killer (NK) cells and T lymphocytes (as reviewed in [7]). An autoimmune-promoting haplotype of SLAM family members containing several polymorphisms within the SLAMF genes has been described in mice [8]. Furthermore SNPs in CD244 and CD229 have been associated with rheumatoid arthritis and SLE disease susceptibility, respectively [9, 10].

Previous studies have documented the altered expression of several SLAM family members in the context of SLE [11–14]. Increased CD229 and CD352 on T lymphocytes from SLE patients was found to promote Th17 differentiation, while loss of T cell CD244 expression is associated with decreased cytotoxic activity [11, 12]. CD244 expression on monocytes and NK cells from SLE patients was also found to be decreased [14]. Finally, increased levels of CD319 have been reported in B cells and, together with an increase in CD229, NK cells and plasmacytoid dendritic cells in patients with SLE [13, 14].

While the above studies have addressed the potential contribution of SLAM family member expression in predominantly lymphocytes and NK cells in SLE, a comprehensive analysis of their expression on myeloid lineages has yet to be addressed. We therefore investigated the cell surface expression of seven SLAM family members on circulating myeloid cells in a cohort of SLE patients in South East Asia. SLAM family receptor expression in patients was compared to levels in healthy controls (HC) and correlated with measures of SLE disease including SLE-related disease activity and serum autoantibody levels.

## Materials and methods

### SLE patients and serology

Thirty-nine adult patients fulfilling the American College of Rheumatology classification criteria for SLE were recruited from the Lupus Clinic at the National University Hospital (NUH), Singapore [1, 2]. Twenty-nine HC matched to the SLE patients for ethnicity and gender were recruited for comparison (control and SLE donors were 19–64 years old) and peripheral blood harvested within an hour of the SLE patient peripheral blood draw. Details of peripheral blood collection and serology analyses for this cohort have been previously described [15]. The study was approved by the local ethics committees. Written informed consent was obtained from all participants prior to recruitment.

### Flow cytometry

Whole blood was stained with directly-conjugated antibodies and incubated for 30 mins at 4 °C. Following red blood cell lysis (BD FACS Lysing solution), myeloid lineages were identified using anti-CD45, anti-CD14, anti-CD11b (BD Pharmingen), anti-CD16 (eBioscience), and anti-CD62L (Beckman Coulter)(online resource 1A, B). For analysis of SLAM family receptor expression, anti-CD150, anti-CD84, anti-CD229 (eBioscience), anti-CD48, anti-CD244, anti-CD352 and anti-CD319 (Biolegend) antibodies were used. Acquisition and analysis were performed using a BD LSRFortessa™ and FlowJo v10.1 for Windows, respectively (Treestar). Median fluorescence intensity (MFI) was normalised to a mean equivalent of fluorochrome (MEF) SPHERO™ Rainbow Calibration Particles and the isotype control, as per the manufacturer’s instructions (online resource 1C).

### Statistics

For flow cytometry, mean equivalent of fluorochrome (MEF) values are reported. Results are expressed as the arithmetic mean ± standard error of the mean (SEM) for a given number of values (*n*). Comparisons between SLE patients and HC were performed using the Mann-Whitney *U* test. For data analysis involving more than two groups, the Kruskal-Wallis non-parametric ANOVA followed by Dunn’s multiple comparison test was used. The Spearman rank correlation was performed for correlation analyses. Statistics and graphs were generated using GraphPad Prism v6.00 for Windows (GraphPad Software).

## Results

Patient demographics are summarised in online resource 2, reproduced with permission from *Rheumatology* [15]. All patients included in the study were positive for ANAs and we observed significant negative correlations between serum complement C3 and C4 levels and anti-dsDNA and anti-snRNP autoantibodies, respectively (online resource 1), in the sera of SLE patients.

CD244 and CD84 were both determined to be broadly expressed on myeloid lineages in SLE patients and HC, while CD48, the ligand for CD244, is expressed only on monocytes (Table 1). In contrast, in both patients and HC, expression of CD352 and CD319 was found to be restricted to eosinophils and monocytes, respectively (Table 1). While prior studies have demonstrated that Ly108, the murine homologue of CD352, is expressed on polymorphonuclear cells (PMNs), we did not detect expression above levels of background isotype staining (data not shown) [16]. Cell surface expression of CD229 and CD150 on circulating myeloid cells was undetected in both patients and HC, potentially reflecting restricted expression on NK cells, lymphocytes and antigen presenting cells (APCs) (data not shown) [13, 17, 18]. CD150 expression on platelets has been previously described however, and this discrepancy is likely attributable to the different antibody clone used in this study [19].

**Table 1:** Expression of SLAMF receptors on myeloid lineages in SLE patients and healthy controls

	SLE patients mean ± SEM (*n*)	Controls mean ± SEM (*n*)
CD48 (SLAMF2)		
Monocytes (MEF)	1180 ± 88 (*37*)	1105 ± 76 (*27*)
CD244 (SLAMF4)		
Monocytes (MEF)	13,547 ± 1464 (*37*)	17,522 ± 1775 (*27*)
Eosinophils (MEF)	14,751 ± 1510 (*29*)	12,253 ± 1176 (*27*)
Platelets (MEF)	1471 ± 135 (*37*)	2034 ± 183 (*27*)
CD84 (SLAMF5)		
PMNS (%)	33.9 ± 3.6 (*39)*	31.6 ± 4.0 (*29*)
Monocytes (MEF)	1509 ± 137 (*35*)	1555 ± 203 (*25*)
Eosinophils (MEF)	745 ± 67 (*27*)	740 ± 105 (*25*)
Platelets (MEF)	841 ± 78 *(35)*	1087 ± 102 (*25*)
CD352 (SLAMF6, NTB-A)		
Eosinophils (MEF)	2262 ± 326 (*18*)	1979 ± 235 (*16*)
CD319 (SLAMF7, CRACC, CS1)		
Monocytes (MEF)	460 ± 152 (*37*)	309 ± 47 (*27*)

*MEF* mean equivalent of fluorochrome, *SEM* standard error of the mean, *n* number of individuals

**Fig. 1:**
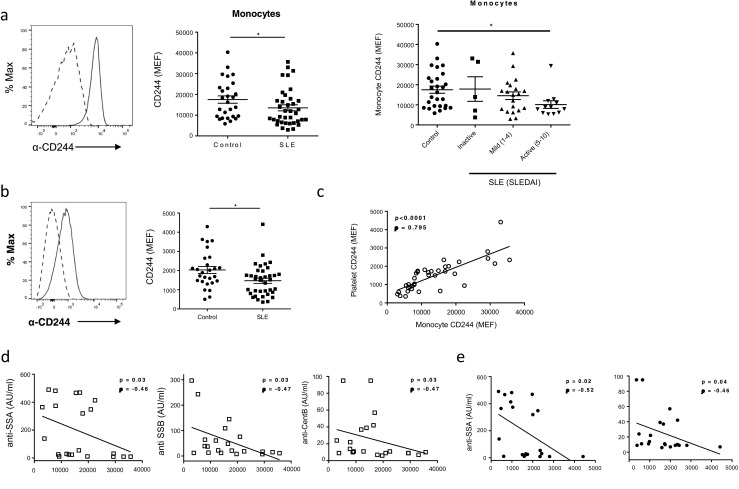
CD244 expression is decreased on monocytes and platelets in SLE patients and correlates with serum autoantibodies. **a** CD244 monocyte expression levels are decreased in SLE patients compared to healthy controls. Patients with active disease show lower levels of CD244. Histogram illustrates anti-CD244 staining on monocytes (*dashed line* represents isotype control). **b** Decreased platelet CD244 expression in SLE patients. Histogram illustrates anti-CD244 staining on platelets (*dashed line* represents isotype control). **c** Decreased levels of CD244 on SLE patient platelets correlate with decreased monocyte CD244 expression. **d** Significant correlations between SLE patient monocyte CD244 expression levels and serum autoantibodies. **e** Significant correlations between SLE patient platelet CD244 expression levels and serum autoantibodies. *Bars* represent mean ± SEM. *P ˂ 0.05

In agreement with a study by Kim et al., levels of CD244 on SLE patient monocytes were significantly lower as compared to HC (Fig. 1a) [14]. Furthermore, SLE patients with active disease were also found to express the lowest levels of CD244 on monocytes. Decreased CD244 expression on platelets from SLE patients was also observed and correlated strongly with decreased monocyte CD244 expression (Fig. 1b–c). While CD244 expression on platelets was noted to be approximately tenfold lower than for monocytes, expression of this SLAM family receptor in this cell type has been reported previously at the RNA level [19].

Our analysis of monocyte and platelet CD244 revealed significant correlations between decreased SLE patient CD244 expression and increased serum autoantibody levels (anti-SSA 52/60, anti-SSB and anti-CentB) (Fig. 1d, e and Table 2).

**Table 2:** Association of CD244 with autoantibody titres

Autoantibody	Monocyte CD244		Platelet CD244	
	*p*	ρ	*p*	ρ
Anti-SSB	*0.033*	−0.468	0.172	−0.310
Anti-SSA60	*0.034*	−0.464	*0.016*	−0.518
Anti-SSA52	0.082	−0.389	0.140	−0.333
Anti-Sm	0.179	−0.305	0.750	−0.074
Anti-Scl70	0.085	−0.385	0.159	−0.319
Anti-RNP	0.278	−0.248	0.704	−0.088
Anti-RibP	0.097	−0.371	0.313	−0.231
Anti-Jo1	0.322	−0.227	0.598	−0.122
Anti-CentB	*0.031*	−0.471	*0.043*	−0.445

*p* probability, *ρ* Spearman’s rank correlation

Significance; *p* < 0.05 indicated in italics

Elevated levels of circulating PMNs have been previously reported in SLE; in agreement with this, we found the proportion of PMNs in peripheral blood to be significantly increased in our patient cohort as compared to HC (online resource 2) [18]. Circulating PMNs were found to express only a single SLAM family receptor, CD84, with no detectable difference in expression levels between SLE patients and HC (Table 1).

## Discussion

In this study, we have demonstrated a significant reduction in CD244 expression on SLE patient monocytes and platelets compared to gender- and ethnicity-matched healthy subjects in an Asian cohort. Furthermore, we showed that monocyte CD244 levels correlated in a negative manner with serum autoantibody levels, including anti-SSA52/60, anti-SSB and anti-CentB antibodies.

Altered expression of SLAM family receptors has been reported in several autoimmune disorders, including Crohn’s disease, rheumatoid arthritis and SLE (as reviewed in [20]). While previous studies have focused predominantly on lymphocytes and NK cells, we performed a comprehensive flow cytometric analysis of SLAM family receptor expression on circulating myeloid lineages. CD150 and CD229 were undetectable on circulating myeloid cells, while monocytes were found to express all remaining SLAM family members, except for CD352. Eosinophils were demonstrated to express CD244, CD84 and CD352, and PMNs were found to express only CD84. CD84 expression was also detected on platelets, together with low levels of CD244.

Our findings on reduced monocyte CD244 levels in SLE are consistent with previous data from a mixed African-American, Hispanic and Caucasian population from the USA [14]. Furthermore, these phenotypic associations in patients are supported by murine studies which have shown an association of the CD244/Ly9/Cs1 region with ANA titres in the B6.*Sle1* benign SLE model [21]. In addition, genetic ablation of CD244 on a non-autoimmune B6 background resulted in a breach of self-tolerance in 30% of female mice aged 12 months old. This study, by Sharpe and colleagues, also demonstrated that CD244-deficient mice have higher levels of anti-DNA autoantibodies following a graft-versus-host disease challenge [22].

CD244 is expressed on NK cells, γδT cells, basophils, monocytes, eosinophils and a subset of CD8 cells (reviewed in [23]). Unlike other SLAMF members, which are homotypic, CD244 binds with CD48 to trigger SAP signalling and activation of the CD244 cytoplasmic ITSM domains. Research on immunological functions to date has focused on the complex role of CD244 in enhancing and decreasing NK cell function [24]. However, it appears that the loss of tolerance in the experimental model described above is NK cell-independent [22].

Innate immune mechanisms play a critical role in the development of SLE and disease severity (reviewed in [25]). Under resting conditions, normal monocytes have little capability for antigen presentation; however, in the permitting inflammatory environment, they can differentiate into both macrophages and dendritic cells (DCs). SLE patient monocytes have been shown to have enhanced DC-like function [26]. In contrast, the phagocytic ability of macrophages to engulf apoptotic material is reduced in SLE [27, 28]. The contribution of CD244 to monocyte, DC, or macrophage cell function is unknown and is an area of future investigation.

PMNs are the most abundant cell type in peripheral blood, and we observed a significant increase in the proportion of circulating PMNs in SLE patients when compared to HC. Mice deficient in Ly108 (CD352) exhibit defects in PMN function leading to increased susceptibility to bacterial infection [16]. We only detected SLAMF member CD84 on PMNs in our cohort however and did not find any associations with disease.

In summary, we describe significant decreases in platelet and monocyte CD244 expression in SLE patients which correlates with ANA titre. Investigating the role of monocyte CD244 may prove to be important in mechanisms of tolerance and autoimmunity.

## Electronic supplementary material

ESM 1 (DOCX 186 kb)
