# Supplementary material for: Brief report: Decreased expression of CD244 (SLAMF4) on monocytes and platelets in patients with systemic lupus erythematosus
Source: Clin Rheumatol. 2017 Jun 8;37(3):811–6. doi: 10.1007/s10067-017-3698-2 (PMC5835059; doi:10.1007/s10067-017-3698-2)
Supplement: Supplementary file 1 — (DOCX 186 kb) [file 10067_2017_3698_MOESM1_ESM.docx]

1 (a) Gating strategy to evaluate SLAMF members, such as CD244 on peripheral blood monocytes eosinophils and polymorphonucleocytes (PMN) using FSC and SSC parameters, CD45, CD16 and CD14. (b) Platelet gating on SSC and CD45^-^ revealed ~99% selection based on CD31 and CD41 positivity (c) SPHERO^TM^ Rainbow calibration particles were used in a subset of patients to normalize expression to cytometer settings and isotype values (Control *n=16-27*, SLE *n=18-37 as indicated in table 1*). Expression of CD244 as Mean Equivalent of Fluorochrome (MEF), calculated using a log/log conversion to MEF and eliminating isotype values as background according to manufacturer’s instructions. (d) Significant correlations are observed in SLE patients with anti-dsDNA and anti-snRNP autoantibodies and decreasing levels of serum complement C3 and C4 respectively. (e) SLE patients have more PMNs compared to controls.

**Online Resource 2.** Patient demographic, clinical and treatment characteristics. Reproduced from Thornhill *et al.*, *Rheumatology* 2017[[1](#_ENREF_1)].

|  | SLE Patient Cohort (n=39) |
| --- | --- |
| \| Age, years, median (range) \| \| --- \| \| Female % \| \| Ethnicity % \| \| Chinese \| \| Malay \| \| Indian \| \| Filipino \| \| Glucocorticoid treatment % \| \| Glucocorticoid dose, median (range) \| \| mg/day Prednisolone \| \| Methotrexate % \| \| Hydroxychloroquine % \| \| Azathioprine % \| \| Mycophenolate % \| \| Cyclosporin % \| \| SLEDAI score, median (range) \| \| Renal disease % \| \| CNS disease % \| \| Cutaneous disease % \| \| ANA positive % \| \| Serum Creatinine, median (range)^*^  μmol/L \| | \| 35 (20-64) \| \| --- \| \| 88 \| \|  \| \| 59 \| \| 25.6 \| \| 7.7 \| \| 7.7 \| \| 97 \| \|  \| \| 7 (0-60) \| \| 3 \| \| 87 \| \| 28 \| \| 13 \| \| 5 \| \| 4 (0-16) \| \| 13 \| \| 8 \| \| 8 \| \| 100 \| \|  \|   52 (40-662) |

*Data for 37 patients

SLEDAI, SLE Disease Activity Index; CNS, Central Nervous System; ANA, Antinuclear antibody

1. Thornhill SI, Mak A, Lee B, Lee HY, Poidinger M, Connolly JE, Fairhurst AM (2017) Monocyte Siglec-14 expression is upregulated in patients with systemic lupus erythematosus and correlates with lupus disease activity. Rheumatology (Oxford). doi:kew498 [pii]10.1093/rheumatology/kew498
